# Leisure Engagement: Medical Conditions, Mobility Difficulties, and Activity Limitations—A Later Life Perspective

**DOI:** 10.1155/2015/610154

**Published:** 2015-08-05

**Authors:** Ingeborg Nilsson, Fredrica Nyqvist, Yngve Gustafson, Mikael Nygård

**Affiliations:** ^1^Department of Community Medicine and Rehabilitation, Occupational Therapy, Umeå University, 901 87 Umeå, Sweden; ^2^Umeå University, Ageing and Living Conditions Program, 901 87 Umeå, Sweden; ^3^Faculty of Education and Welfare Studies, Social Policy Unit, Åbo Akademi University, 651 01 Vasa, Finland; ^4^Department of Community Medicine and Rehabilitation, Geriatric Medicine, Umeå University, 901 87 Umeå, Sweden

## Abstract

*Objectives*. This study aims to investigate the impact of medical conditions, mobility difficulties, and activity limitations on older people's engagement in leisure activities. *Methods*. The analyses are based on a cross regional survey carried out in 2010 in the Bothnia region (Northern Sweden and Western Finland). A posted questionnaire, which included questions on different aspects of leisure engagement, medical history, and health, was sent out to older persons in the region. The final sample consisted of 5435 persons aged 65, 70, 75, and 80 years. The data was analyzed by using ordinary least squares (OLS) multivariate regression. *Results*. The most important predictor of leisure engagement abstention among older people is the prevalence of activity limitations, whereas mobility difficulties and medical conditions play less important roles. The strong negative association between activity limitations and leisure engagement remains significant even after we control for individual, sociodemographic characteristics, and country. *Discussion*. This study provides a window into leisure engagement in later life and factors influencing the magnitude of engagement in leisure activities.

## 1. Introduction

Older people's participation in leisure activities, such as involvement in cultural, social, and physical activities, is known to be positively associated with health and survival [1]. It is therefore important to improve the understanding of the risk factors that might cause a decline in such participation. Accordingly, this study aims to investigate the impact that health-related risk factors, such as medical conditions, mobility difficulties, and activity limitations, may have on older people's engagement in leisure activities.

European countries are currently facing major demographic changes due to substantial increases in longevity (a large reduction in late life mortality) and declines in fertility [2, 3]. As a consequence, the number and proportion of older people will increase. This fact has led to a growing interest in understanding how to meet the needs of an ageing population. It is also a public health priority that includes, among other things, the identification of components that can promote factors to support active and healthy ageing [4, 5]. In an aging population, the prevalence of health-related risk factors such as medical conditions, mobility related difficulties (limits in functions), and activity limitations (inability to perform activities) increase with age [6]. The relation between the above-mentioned health-related risk factors and health is, however, complex and much discussed [7]. According to the World Health Organization, health is a state of complete physical, mental, and social well-being, and to reach this state we must be able to identify and realize aspirations in life, satisfy our needs, and be able to change or cope with the environment [8]. Therefore, the relationship between engaging in activities and becoming healthy is an important perspective of health [9] with a long history [10].

On a general level, engagement in leisure activities has demonstrated positive health-related outcomes [11] and correlates explicitly with increased survival and life expectancies [12], lower mortality rates [13–17], and higher levels of happiness [18, 19]. Engagement in leisure activities seems therefore to be an important health promoter in the case of older people [17]. Engagement and participation in leisure activities are reported slowly decline over time in later life [20, 21], but how this decline can be promoted is less known. Participation in leisure activities is often studied from the aspect of performing activities as such, while some studies also highlight the importance of incorporating the purpose or motivational aspects for added understanding [22, 23]. Being motivated is a crucial component for making the leisure activities meaningful [11], and therefore the motivation component is an important factor to take into account when conducting research on leisure engagement and its relation to health-related conditions.

Earlier research has shown that functional decline could be an important indicator of ill-health in later life [24, 25]. However, according to the World Health Organization [26], functional decline and limitations might not be the direct cause of the limited ability to perform activities, as the adaptation to the environment or by the person could reduce the negative effect of such decline. Hence, understanding the cause to activity limitations is complex; Wu et al. [27], for example, did not find any associations between medical diseases and activities of daily living (ADL), whereas Gill et al. [24] found change in physical performance as independently associated with ADL dependency. While maintaining healthy habits is described as preventing the deterioration of functional capacity [28, 29], few studies focus on leisure engagement as a result or indicator of a person enjoying good health. Atchley [30] found that limitations in performance affected leisure patterns in older people, but if and how this is true also for mobility difficulties and other important health-related risk factors in later life needs further investigation.

In this study, we therefore wanted to explore potential health-related risk factors related to leisure engagement decline by studying the impact of medical conditions, mobility difficulties, and activity limitations in relation to leisure engagement. While studying this relation, it is important to consider the influence of personal characteristics upon these relationships. For example, it is known that later life health problems seem to vary between genders [31], to be influenced by socioeconomic aspects, and to vary between countries or geographic regions [32]. Therefore, these potential confounders must be considered as having a possible impact on this relation. Specifically, the research questions for this paper are the following.

1. Is there a relationship between medical conditions, mobility difficulties, activity limitations, and leisure engagement in older people?
2. Is the relationship influenced by sociodemographic aspects such as gender, age, economy, and geography?

## 2. Data and Methods

### 2.1. Sample

The analyses are based on a cross regional survey carried out in 2010 as a part of an interregional EU-funded research project (Gerontological Regional Database and Resource Centre, GERDA). The overall aim of the multidisciplinary project was to map living and health conditions of older adults (aged 65, 70, 75, and 80 years) in the Bothnia region, that is, on both sides of the* Gulf of Bothnia*, in* Västerbotten* in Sweden and in* Österbotten/Pohjanmaa* in Finland (more information about the project is available at the project website (http://web.novia.fi/gerda/)). Although the two regions* Österbotten* and* Pohjanmaa* belong to the same geographical region, they can in fact be treated as two separate regions due to different linguistic conditions (the (technical) division between* Österbotten* and* Pohjanmaa* relates to a language stratification of citizens in this particular West-Finnish region. Elderly Swedish-speaking inhabitants were coded as belonging to* Österbotten *and those with Finnish as their mother tongue were coded as belonging to* Pohjanmaa*). In this paper, however, we do not separate these two areas from each other, since the linguistic characteristics of the Finnish population are controlled for by the language variable.

In 2010, the Swedish region* Västerbotten* consisted of 15 municipalities, including two more densely populated areas (*Umeå* and* Skellefteå*), with an overall population of approximately 260,000 inhabitants. The overall population in the West-Finnish region* Österbotten/Pohjanmaa* (including the town of Vaasa) consisted of approximately 178,000 inhabitants [33]. Although the above-mentioned regions share several common structural features, such as common cultural characteristics and common historical bonds, there are also noticeable differences between them, such as differing linguistic conditions. Finland is an officially bilingual state with a large Finnish-speaking majority and a small Swedish-speaking minority of approximately 6 percent. However, in* Österbotten/Pohjanmaa,* 51 percent of the population belong to the Swedish-speaking group and form to some extent a majority at the local level. In 2010, three out of 17 municipalities in* Österbotten/Pohjanmaa* were officially monolingual (Finnish) whereas Swedish-speakers formed the local majority in nine municipalities. The two language groups in Finland were sent questionnaires in their own language.

A total sample of 10,696 was selected from the National Tax Board in Sweden and the Population Register Centre in Finland. Questionnaires were sent to all people that in 2010 were 65, 70, 75, and 80 years old in rural municipalities, to every second person in the most populous town in Finland and to every third person residing in the two most populous towns in* Västerbotten*. In total, 6 838 persons (64%) replied. The response rate varied between the regions, with 70% responding in* Västerbotten*, 62% in* Österbotten,* and 53% in* Pohjanmaa*. The response rate decreased marginally with age. The response rate was somewhat higher amongst the two younger age groups (66%) than those aged 75 and 80 years (61.9 and 59.2%, resp.).

In order to be selected as a part of the sample, valid responses on leisure engagement were required. This criterion narrowed the final sample down to a total of 5435 older persons. A description of the sample is found in Table 1.

### 2.2. Data Collection

A posted questionnaire was sent out during late 2010 and included a broad range of questions related to aspects of societal engagement, medical history, health, and sociodemography. The battery of questions was developed by the multidisciplinary team of researchers included in the GERDA, and for this paper we analyzed medical conditions (integrating ≥5 pharmaceutical drugs, stroke, heart disease, cancer, and hospital stays during the last 12 months into an index); mobility difficulties (integrating fear of falling and mobility device into an index); activity limitations (integrating independent bathing and independent cleaning into an index); and leisure engagement. The sociodemographic variables included in the analysis were age (65/70/75/80 yrs), language (Swedish/Finnish and other languages), gender (man/women), civil status (single/together), income (≤1000*€*/>1000*€*), education (≤9 yrs/≥10 yrs), and country (Finland/Sweden).

Leisure engagement was measured by asking about two aspects of 20 different leisure activities (task): first if the participant had a habit of performing the task and then if the participant was motivated to perform the said task. These questions were a part of the MNPS leisure checklist that has been used in previous similar samples and that has been evaluated for its validity [23, 34].

### 2.3. Data Analysis

Firstly, we calculated the extension of medical conditions, mobility difficulties, and activity limitations in every person by adding up each component in the index into a number. More specifically, each person was assigned a number that put their medical condition between 0 and 5 based on if they reported using ≥5 pharmaceutical drugs, personal incidence of stroke, heart disease, cancer, or/and hospital stays during the last 12 months. Similarly, a number between 0 and 2 was assigned for mobility difficulties (adding a fear of falling, dependency on mobility devices) and activity limitations (dependency on help for bathing, dependency on help for cleaning). A higher number was interpreted as showing a more severe medical condition, mobility difficulties, or activity limitations.

Secondly, to generate the measures of leisure engagement, the raw data of leisure performance and the raw data of leisure motivation were combined and subjected to the Rasch rating scale analysis by using the WINSTEPS program [35]. This procedure has been used and found to be a valid measurement for groups of older people [36]. The generated data was treated as valid if it met the common criteria for surveys of MnSq ≤ 1.5 and *z* ≤ 2.0 [37]. To be included in the study, a valid response regarding leisure engagement was required. In total, data from 1 403 respondents was excluded due to invalid responses. A major reason for this error was that many participants had not answered the question about leisure motivation (“do you want to perform this activity?”).

Lastly, we exported the leisure engagement scale measures to IBM SPSS Statistics, version 20, for continued analysis. The relationship between the engagement in leisure activities of older adults, different health indicators and sociodemographic control variables was assessed by using ordinary least squares (OLS) multivariate regression. We used the above-mentioned measure for leisure engagement as our dependent variable as well as three indexes measuring medical condition, mobility difficulties, and activity limitations as independent variables. We also controlled for sociodemographic aspects. Three regression models were calculated. The first model calculated a bivariate regression coefficient for each separate health indicator index on leisure engagement. The second model consisted of multivariate regressions of the above-mentioned health indicator indexes on leisure engagement. The third model was a full model assessing the association between leisure engagement and health indicators while controlling for sociodemographic characteristics.

## 3. Results

In this study a total of 5435 participants met the inclusion criteria, 1375 from Österbotten (25.3%), 845 from Pohjanmaa (15.5%), and 3215 from Västerbotten (59.2%). The most dominant type of person found in the sample was a 65-year-old (40.2%) women (55.5%) living in Västerbotten, Sweden (59.2%), together with someone (74%) and earning more than 1000*€* a month (72.8%). Details about the sample are described in Table 1. The basic characteristics were similarly distributed in Finland and Sweden. Of the total sample 59.2% were Swedes (from Västerbotten, Sweden), 25.3% were Swedish-speaking Finns (from Österbotten, Finland) and 15.5% were Finnish-speaking Finns (from Pohjanmaa, Finland). Leisure engagement varied in the sample between the most engaged (5.10) and the least engaged (−4.60) with a mean 0.28 (SD 0.99).

Every fifth participant (about 20%) used 5 or more pharmaceutical drugs and reported a hospital stay during the last 12 months. The most frequently reported medical diagnosis was (some form of) cancer (15%). One quarter of the participants (about 25%) reported a fear of falling and about 14% needed help with cleaning. More details about medical diagnoses, mobility difficulties, and activity limitations are described in Table 2.

**Figure 1:**
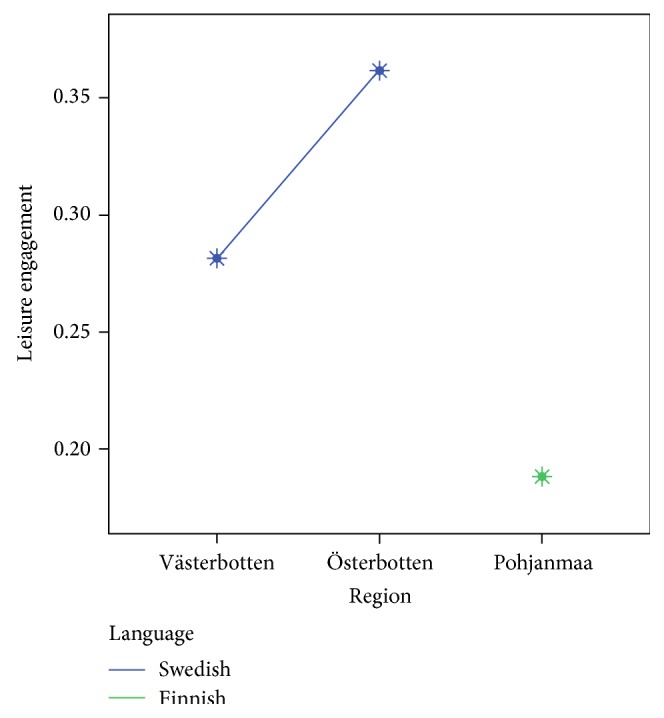
Illustration of how leisure engagement varies between Swedish- and Finnish-speakers in the study.

The first two models of the OLS regression (see Table 3) indicate that medical conditions, mobility difficulties, and activity limitations all have hampering effects on the leisure engagement of older adults, both when we consider the bivariate associations between each of the three indicators and leisure engagement (model 1) and when their relative importance for leisure engagement (model 2) is taken into consideration. As is shown in the table, the indicator having the biggest impact on the leisure engagement of older adults is activity limitations, while the two other health status indexes play somewhat lesser roles. Interestingly, the strong negative association between activity limitations and leisure engagement remains significant (which is also the case with mobility difficulties) even after we control for individual, sociodemographic characteristics, and country (model 3). This suggests an independent association between activity limitations, mobility difficulties, and leisure engagement. The table also shows that leisure engagement tends to decline with old age and that leisure engagement is higher among Swedish-speaking older adults than among Finnish-speakers and persons with other mother tongues. It is intriguing to note that one's mother tongue seems to play an important role in this respect, although the country variable does not seem to matter a great deal. As it is revealed in the figure, the Swedish-speakers in Finland also show a high rate of leisure engagement, which may explain why the country variable remains insignificant (see Figure 1). Furthermore, Table 3 shows that leisure engagement is higher among women, persons living together with someone else, persons with high incomes (pensions), and persons with higher levels of education.

## 4. Discussion

In this paper, we have analyzed the relationship between leisure engagement and medical conditions, mobility difficulties, and activity limitations. This study demonstrates that having a fear of falling, using mobility devices, and needing help with bathing and/or cleaning all have a significant impact on the level of leisure engagement in the case of older people. This impact remains significant even after controlling for variables such as gender, age, civil status, income, education, and language group affiliation.

For the purpose of this study, we operationalized mobility difficulties and activity limitations as an index that assessed whether older persons were experiencing a fear of falling and using mobility devices or if they were in need of help in order to bathe or clean themselves, respectively. Even though this is only one way of operationalizing mobility difficulties and activity limitations, it still gives an insight into how mobility issues as well as ability factors influence the extent to which older persons engage in leisure activities. Mobility issues and particularly a fear of falling have previously been studied and found to influence physical leisure activities negatively [38, 39].

Medical conditions also play a role in predicting the level of leisure engagement. Yet this variable does not seem to play a role as big as that of mobility difficulties and activity limitations. Moreover, the variable of medical conditions loses its strength after controlling for sociodemographic variables. These results are somewhat supported by earlier findings [6, 40], which report that older people may view themselves as healthy despite suffering from chronic illnesses and disabilities. However, the negative link between illnesses or diagnoses and activities is often taken for granted or regarded as obvious (e.g., [41–43]) even though other researchers found that engagement in activities is affected by more than just diseases. Life style factors as well as persons' physical and social environment, for example, have been noted to play a role in the level of engagement in such activities [44–47].

With a regression model explaining about 10% of the variance in leisure engagement, this study demonstrates the need to examine leisure engagement no matter medical conditions, mobility difficulties, or activity limitations. Moreover, this study supports the need to consider age, gender, civil status, and socioeconomic status in terms of income and educational level when examining leisure engagement in the case of older people. In this study, leisure engagement varied between Finnish- and Swedish-speakers, but the country variable as such showed no impact on their engagement in leisure activities. This is an interesting finding that seems to separate the effects of language group affiliation from those of the geographic region where a person is resident (e.g., [32]). The results found in this paper also corroborate the findings from a recent study, which found that good self-rated health was high among Swedish-speakers in Sweden and Finland, respectively, but lower among Finnish-speakers in Finland [48]. Further, being in line with previous research [49], it seems like historical, social, and cultural differences attributed to belonging to a specific language group in Finland are playing an important role in explaining language group differences in leisure engagement. It has been suggested that the Swedish-speaking community in Finland live in tighter social networks as compared to the Finnish-speaking community [49] which might explain the high levels of leisure engagement among the Swedish-speakers in Finland in our study.

To what extent does leisure engagement of older persons depend on their level of activity limitations, their level of mobility difficulties, or their medical diagnoses, and to what may such engagement be related to other factors not examined in the present study? These factors need to be identified and highlighted in future studies. Environmental issues and physical and social factors may also be important here, as suggested in many theories [50–52]. Supports and barriers in the social, physical, or societal environment could, for example, be a part of interventions [53] but are also shown to influence physical activities and suggested to be used as predicting factors [54]. There might also be other, yet unknown, factors of importance to identify and incorporate into future evaluations of leisure engagement.

Earlier research supports a relationship between self-rated health, seen as an overall measure of health, and leisure engagement [55, 56] and this needs to be investigated further. Perhaps self-rated health could go together with medical conditions, mobility difficulties, and activity limitations to create a broader understanding of factors influencing the engagement in leisure activities.

Based on the results, we can question whether medical conditions have a great impact on leisure engagement. Other findings similarly reveal limitations in using medical conditions to predict a decline in the activities of daily living [57]. Hence, it is important to remember that only a few diagnoses and signs of medical conditions were included in this study's medical condition variable. If other diagnoses or signs, such as symptoms of depression and cognitive limitations, were included, the predictive strength of the regression models might be improved in terms of leisure engagement. Another limitation of this study is that self-reports of diseases are prone to be influenced by individual bias, particularly in the case of illnesses perceived as nonthreatening and that do not hamper a person's ability to live normally [58]. These illnesses may nevertheless increase the risks for a decline of a person's ability to function normally and need therefore to be considered. Although we used a population-based cohort, the exclusion from the analyses of participants with invalid responses on leisure engagement measures may have introduced bias and reduced the generalizability of the results. However, as the study is not limited to include only the performance of leisure activities, but also the motivational aspect of these activities, our measure on leisure engagement can arguably be seen as robust.

This study is based on self-reports, that is, on the accounts of older people assessing their engagement in leisure activities as well as their experienced medical diagnoses, mobility difficulties, and activity limitations. Together, this contributes to an understanding about leisure engagement in later life. There is however a great need to study also other variables in the complex field of leisure engagement.

## Figures and Tables

**Table 1:** Frequencies (%) of basic characteristics in the studied sample.

	Total sample *n* = 5435	Finland *n* = 2220	Sweden *n* = 3215
Age			
65	2185 (40.2)	965 (43.5)	1220 (37.9)
70	1357 (25.0)	508 (22.9)	849 (26.4)
75	1091 (20.1)	449 (20.2)	643 (20.0)
80	800 (14.7)	298 (13.4)	502 (15.6)
Gender			
Women	3018 (55.5)	1259 (56.7)	1759 (54.7)
Men	2416 (45.5)	961 (43.3)	1455 (45.3)
Living condition			
Single living	1401 (26.0)	517 (23.5)	884 (27.8)
Living together	3983 (74.0)	1686 (76.5)	2297 (72.2)
Education level			
Shorter (up to 9 yrs)	2469 (46.4)	925 (42.3)	1544 (49.1)
Longer (10 yrs or more)	2857 (53.6)	1255 (57.6)	1602 (50.9)
Income level			
≤1000€	1384 (27.2)	599 (28.3)	786 (26.5)
>1000€	3700 (72.8)	1515 (71.7)	2185 (73.6)
Leisure engagement			
M (SD)	0.28 (0.99)	0.29 (0.97)	0.27 (1.0)
Range	−4.90–5.10	−4.90–5.0	−4.60–5.10

**Table 2:** Frequencies (%) of medical conditions, mobility difficulties, and activity limitations in the studied group.

	Total sample *n* = 5435	Finland *n* = 2220	Sweden *n* = 3215
Medical conditions			
≥5 pharmaceutical drugs	1074 (20.4)	370 (17.2)	704 (22.5)
Stroke	352 (6.9%)	100 (4.8)	252 (8.2)
Heart disease	435 (8.7)	147 (7.2)	288 (9.7)
Cancer	749 (14.6)	299 (14.4)	450 (14.7)
Hospital care during the last 12 months	1018 (19.3)	409 (19.0)	609 (19.5)
Mobility difficulties			
Mobility device	645 (12.2)	228 (10.5)	417 (13.3)
Fear of falling	1389 (26.4)	454 (21.1)	935 (30.1)
Activity limitations			
Need help with bathing	326 (6.1)	107 (4.9)	219 (6.9)
Need help with cleaning	774 (14.4)	323 (14.8)	451 (14.2)

**Table 3:** The effects of medical conditions, mobility difficulties, and activity limitations on the leisure engagement of older adults. Results from bivariate and multivariate OLS analyses.

Independent variables	Model 1	Model 2	Model 3
Health-related risk factors			
Medical conditions (index)	−0.134^***^	−0.052^***^	−0.036^*^
Mobility difficulties (index)	−0.273^***^	−0.115^***^	−0.079^***^
Activity limitations (index)	−0.504^***^	−0.196^***^	−0.168^***^
Sociodemographic variables			
Age (cont.)			−0.104^***^
Language: Swedish (ref. Finnish and others)			0.068^***^
Gender: female (ref. male)			0.056^***^
Civil status: partnership (ref. single)			0.052^***^
Income: high (ref. low)			0.062^***^
Education: high (ref. low education)			0.083^***^
Country: Sweden (ref. Finland)			−0.015

*n*		5435	5435
Adjusted *R* square		0.071	0.101

Note: the first model shows nonstandardised Beta coefficients from bivariate regressions of each health indicator of leisure engagement, whereas models 2 and 3 show standardised Beta coefficients from multivariate regressions. The variables, medical conditions, mobility difficulties, and activity limitations, are indexes. Age is a continuous variable and the other variables are “dummy” variables. ^*^ *P* < 0.95 and ^***^ *P* < 0.999.
